# Brittle fracture studied by ultra-high-speed synchrotron X-ray diffraction imaging

**DOI:** 10.1107/S1600576722006537

**Published:** 2022-07-30

**Authors:** Antoine Petit, Sylvia Pokam, Frederic Mazen, Samuel Tardif, Didier Landru, Oleg Kononchuk, Nadia Ben Mohamed, Margie P. Olbinado, Alexander Rack, Francois Rieutord

**Affiliations:** a Univ. Grenoble Alpes, CEA, LETI, MINATEC Campus, F-38054 Grenoble, France; b Univ. Grenoble Alpes, CEA, IRIG-MEM-NRS, F-38000 Grenoble, France; c SOITEC, Parc Technologique des Fontaines, 38190 Bernin, France; d ESRF – The European Synchrotron, 71 avenue des Martyrs, F-38043 Grenoble, France; Australian Synchrotron, ANSTO, Australia

**Keywords:** X-ray diffraction, ion implantation, crack-front shape

## Abstract

*In situ* investigations of cracks propagating at up to 2.5 km s^−1^ along an (001) plane of a silicon single crystal are reported, using X-ray diffraction megahertz imaging with intense and time-structured synchrotron radiation. The studied system is based on the Smart Cut process, where a buried layer in a material (typically Si) is weakened by microcracks and then used to drive a macroscopic crack (10^−1^ m) in a plane parallel to the surface with minimal deviation (10^−9^ m). A direct confirmation that the shape of the crack front is not affected by the distribution of the microcracks is provided. Instantaneous crack velocities over the centimetre-wide field of view were measured and showed an effect of local heating by the X-ray beam. The post-crack movements of the separated wafer parts could also be observed and explained using pneumatics and elasticity. A comprehensive view of controlled fracture propagation in a crystalline material is provided, paving the way for the *in situ* measurement of ultra-fast strain field propagation.

## Abstract 2

Crack propagation in a silicon single-crystal wafer is tracked *in situ* using synchrotron-based ultra-high speed X-ray diffraction imaging. The high spatio-temporal resolution reached in diffraction imaging mode allows for assessing different parameters such as crack velocity or post crack movements of the separated wafers.

## 1. Introduction

Ever since the very first human-made knapped tools, the control of fracture propagation in brittle materials has been a driver of technological development. Nowadays, a broad range of applications rely on crack propagation control, from the mitigation of damage, *e.g.* from impacts in glass screens or vehicle windscreens, to industrial processes harnessing fracture to achieve clean cuts over large distances. Frequently, experimental *in situ* studies present a challenge, since cracks can propagate at up to a few kilometres per second, in particular in opaque materials, and one is often limited to a *post mortem* analysis (Ravi-Chandar, 1998 ▸). The spatial extent of a crack in a brittle material is small, with very rapidly varying strain and stress fields near the crack tip position. In addition, the crack propagation speed is very high, typically of the order of the Rayleigh wave speed or the speed of sound in the material (several kilometres per second). Hence, obtaining a submillimetre resolution without motion blur requires exposure times much shorter than a microsecond. These stringent requirements can only be met using bright synchrotron radiation: the pulsed source enables snapshot images with sub-nanosecond exposure time and hence freezes ultra-fast motion in the images.

X-ray scattering, and in particular X-ray diffraction, is a powerful technique whenever crystalline materials are to be studied (Huang *et al.*, 2016 ▸). Diffraction-based imaging, *i.e.* X-ray (Bragg) topography, takes advantage of the contrast stemming from defects in single crystals to image them in real space, yielding quantitative information, as recently reviewed by Danilewsky (2020 ▸). In the field of fracture mechanics, X-ray topography has proven to be an efficient tool to investigate cracks in single crystals (Tanner *et al.*, 2012 ▸, 2013 ▸, 2015 ▸; Danilewsky *et al.*, 2013 ▸; Atrash *et al.*, 2017 ▸). Numerous studies have been conducted to measure the strain and estimate the stress near crack tips, but they have usually only dealt with static or quasi-static distributions of strain and stress, as discussed in an extensive review (Withers, 2015 ▸). Combined with hard synchrotron radiation, X-ray topography can be performed in real time (Tuomi *et al.*, 1983 ▸; Rack *et al.*, 2010 ▸) under mechanical (Tanner *et al.*, 2017 ▸; Tsoutsouva *et al.*, 2021 ▸) or thermal loading (Danilewsky *et al.*, 2011 ▸, 2013 ▸). More recently, some of the present authors have shown that thermally induced crack propagation could be followed *in situ* using X-ray imaging, in both transmission and diffraction conditions, with peak speeds observed of the order of a few metres per second (Rack *et al.*, 2016 ▸). We show in this report that synchrotron X-ray diffraction imaging can actually be fast enough for real-time and real-space study of fracture mechanics near the speed of sound, providing unprecedented direct observations of crack propagation and a wealth of information at different time and length scales that are otherwise inaccessible.

This report focuses on single-crystal silicon, the most common substrate for the microelectronics industry. In particular, our test vehicles are assemblies of silicon wafers prepared with the Smart Cut technology. This process is widely used to transfer thin single-crystal layers to a different substrate by fracturing an implanted interface in a controlled manner (Bruel, 1995 ▸). The transfer can be done on any substrate, and this technology is currently used for the mass production of silicon-on-insulator substrates (Di Cioccio *et al.*, 1997 ▸; Tauzin *et al.*, 2005 ▸). The full workflow of the technology is described elsewhere and can be summarized as follows. First, a high dose of light ions, typically hydrogen (Bruel, 1995 ▸; Aspar *et al.*, 1997 ▸) or a mixture of hydrogen and helium (Agarwal *et al.*, 1998 ▸; Duo *et al.*, 2000 ▸), is implanted in a silicon substrate. This results in the formation of a buried weakened layer within the substrate. By bonding a stiff substrate (termed an ‘acceptor’ wafer) onto the surface of the implanted wafer, the implantation-related defects grow in plane under annealing, forming a network of pressurized microcracks (Moriceau *et al.*, 2012 ▸; Claverie *et al.*, 2018 ▸). The size of these microcracks depends on the annealing conditions and the nature and amount of implanted gases. Once the surface coverage of the microcracks is large enough, a manually generated or naturally occurring fracture propagates through the weakened layer and effectively transfers the surface film to the acceptor (Bruel, 1995 ▸).

Before the fracture, the system is in a metastable state where the interface is internally loaded by the distribution of pressurized microcracks. Each microcrack is in pseudo-equilibrium, as its evolution is very slow, and the equilibrium state can be described using the standard Griffith argument, where the energy release rate is equal to the surface energy (Penot *et al.*, 2013 ▸). The internal load on the microcrack faces is due to the inner pressurized gas which has precipitated from the implanted material layer. The pressure load is balanced by the elastic deformation of the material and the surface opening. The implanted dose is usually around 10^16^ at. cm^−2^. Therefore the pressure in a micrometre-sized crack is of the order of 100 MPa and the corresponding microcrack height is in the nanometre range. A crude description of each microcrack is the penny-shaped crack model that can be found in standard textbooks (Tada *et al.*, 1985 ▸).

Upon fracture, the crack front propagates in the microcrack layer, akin to a two-dimensional pre-perforated mater­ial. The crack propagation plane is close to the middle of the 2 × 775 µm thick silicon wafer assembly, parallel to the wafer external surfaces, *i.e.* in the strip geometry. It has been shown that in this case, the crack velocity rapidly reaches an asymptotic velocity that is a fraction of the Rayleigh velocity (Marder, 1991 ▸), which prevents a deflection of the front (Kermode *et al.*, 2008 ▸). The crack front emits acoustic waves and the feedback of those acoustic waves on the propagating crack front causes small deviations of the fracture plane (Massy *et al.*, 2015 ▸; Landru *et al.*, 2021 ▸). This translates into periodic patterns of roughness modulations on the surfaces obtained after splitting (Massy *et al.*, 2015 ▸, 2018 ▸). Similar results have also been found in asperity-free silicon samples (Wang *et al.*, 2020 ▸). Therefore, the study of crack propagation is key to understanding dynamic brittle fracture in such structures and its impact on the morphology of the separated surfaces.

As mentioned earlier, the *in situ* experimental study of crack propagation is difficult in opaque brittle materials. Infrared (IR) lasers have been used to measure the crack velocity (Massy *et al.*, 2015 ▸) and crack opening displacement (Massy *et al.*, 2018 ▸), but this technique only gives a point measurement at the laser spot position. To the best of our knowledge, no direct imaging of crack front propagation at Rayleigh speed in silicon substrates has been reported so far. Here, we fill this gap by presenting an original approach taking advantage of short X-ray synchrotron pulses to illuminate the Si crystal in the diffraction condition and using an ultra-high-speed X-ray detector to image the crack at different steps of its propagation. Our direct observation of crack propagation provides measurements of both the crack front shape and its velocity, together with post-split wafer deformation.

## 2. Methods

### 2.1. Experimental setup

**Figure 1:**
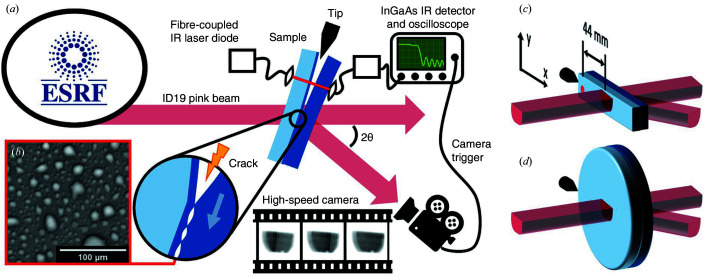
(*a*) Top view of the imaging experiment setup used on beamline ID19. The ID19 pink beam illuminates the sample (bonded wafers) in the diffraction condition, and a high-speed camera connected to an image intensifier records the diffracted image on the scintillator. The motorized tip is used to initiate the fracture mechanically, and this is detected by the IR laser, which in turn is used to trigger the camera. The enlargement at the centre of the sample shows the propagation of the fracture through the layer of pressurized microcracks. (*b*) Top view (IR confocal microscopy image) of the layer of pressurized microcracks made by implantation-related defects after a few hours of annealing. (*c*), (*d*) Three-dimensional representations of the strip and wafer samples, respectively. The red dot represents the IR laser.

For this experiment, ultra-high-speed diffraction imaging was deployed on the ID19 beamline at ESRF, as shown in Fig. 1 ▸(*a*) (Weitkamp *et al.*, 2010 ▸; Escauriza *et al.*, 2018 ▸). The X-ray setup details of the ID19 beamline follow a protocol for high-speed diffraction imaging of wafer fracture (Rack *et al.*, 2016 ▸). The beamline undulators were optimized for 20 keV photon energy. The impinging radiation on the wafer was basically white, with only a diamond and an aluminium attenuator in the beam. We could illuminate a wide area of the sample with a large and parallel beam thanks to the long distance between the source and sample (150 m). The typical beam size at the sample position was a rectangle of 21 × 12.5 mm. For the experiment, the ESRF operated in the timing mode termed the 4-bunch, where four highly populated electron bunches (10 mA) are stored equidistantly in the storage ring (100 ps pulse duration, 704 ns pulse period; Rutherford *et al.*, 2016 ▸).

Two types of samples were used. The starting material was an assembly of two [001]-oriented silicon wafers, 300 mm in diameter and 775 µm thick, prepared by implantation of hydrogen and helium at doses of around 10^16^ at. cm^−2^, direct bonding, and annealing, to form a buried layer of microcracks. A typical image of these cracks obtained by IR confocal microscopy is given in Fig. 1 ▸(*b*), showing an assembly of flat microcracks seen from above with typical sizes in the range of a few micrometres after a few hours of annealing at 648 K. The assembled materials were then either cut along the radius in 20 mm-wide strips, with their length along [110] or [100] [Fig. 1 ▸(*c*)], or used as they were [Fig. 1 ▸(*d*)].

The samples were mounted vertically on a rotation stage such that their [110] or [100] direction (*i.e.* their length for the strip samples) was horizontal. They were then rotated around a vertical axis to bring the (220) or (400) planes into the diffraction condition in the horizontal scattering plane, in the Laue transmission geometry. For the full wafer sample, the 220 reflection was chosen.

Initially, the intensity received on the detector is constant, since the two wafers are still bonded and do not move. When the crack front crosses the beam-illuminated area, the transmitted intensity changes due to the assembly opening following the crack front, thus allowing imaging of the crack front. The scattered X-ray beam image is converted into visible light using a combination of a scintillator screen and an X-ray image intensifier (Ponchut, 2001 ▸). The image was recorded using an ultrafast camera, model HPV-X2 (Shimadzu Corporation, Japan) (Olbinado *et al.*, 2017 ▸), placed 170 cm downstream from the sample, and offset by the scattering angle (18.6° for 220, 26.4° for 400). The camera can acquire 256 frames at a rate of up to 10 MHz and an exposure time down to 50 ns (depending on the acquisition mode). Here, thanks to the stroboscopic nature of the incident radiation, the effective exposure time is given by the bunch length and not by the integration window of the camera, provided that the integration window is equal to or larger than the pulse period (single-bunch imaging). We thus used the camera either in 1 MHz mode or 1.4 MHz mode (integration times of 700 and 400 ns, respectively). The effective pixel size of the detector (camera coupled with X-ray image intensifier) is either 300 or 165 µm, depending on the magnification ratio selected.

The triggering of the camera was performed using the optical setup described below. An IR laser beam is directed to an InGaAs IR photodiode through the sample in such a way that the fracture front crosses the laser beam before the X-ray illuminated area. The photodiode signal is recorded by an oscilloscope and a trigger threshold can be set on the transmitted intensity, as the latter changes sharply when the crack front crosses the laser beam. The time delay between the trigger signal and the camera can be adjusted according to the distance between the IR laser spot and the X-ray illuminated region, and according to the crack velocity. In addition, the crack opening following the crack front creates air wedge interference fringes, which are visible in the transmitted signal from the IR photodiode. These signals can be used for dynamic measurement of the crack velocity and give detailed information on the vibrations of the wafers associated with the crack front propagation, including acoustic (Lamb) waves (Massy *et al.*, 2015 ▸, 2018 ▸). Finally, in our experiment, the samples were pre-annealed to develop microcracks up to the desired microcrack surface coverage, and a motor-driven blade was used for mechanical induction of the fracture at room temperature. The motorized blade was slowly inserted into the bevelled edge of the wafers until the fracture occurred [Fig. 1 ▸(*a*)].

## 3. Results and discussion

### 3.1. Crack front shape

**Figure 2:**
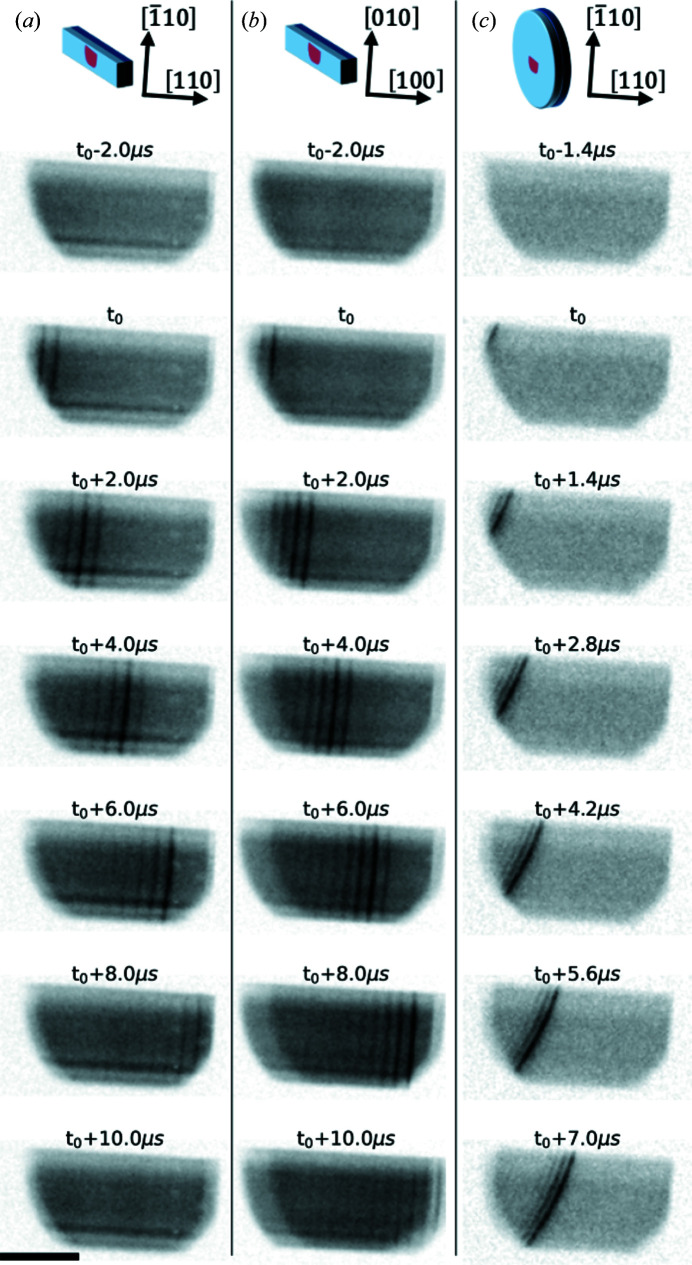
X-ray diffraction imaging of the crack front in (*a*) the silicon [110] strip, (*b*) the silicon [100] strip and (*c*) the full 300 mm silicon wafer assembly. The time *t*_0_ indicates the entry time of the crack front in the field of view. The scale bar is 10 mm.

The evolution of the diffracted signal (220 or 400 Bragg reflection) recorded by the camera when the crack crossed the field of view is shown in Fig. 2 ▸ for the different types of samples. Figs. 2 ▸(*a*) and 2 ▸(*b*) show crack propagation in the strip samples, while Fig. 2 ▸(*c*) shows propagation in a full assembly of 300 mm wafers. For the two types of samples, the first image is taken a few microseconds before the crack front reaches the illuminated area. The following images are then taken at regular intervals in a stroboscopic manner (for clarity, we only show every other image). A comparison of the different delayed images shows the crack front propagation. As we will show below, the enhanced scattered intensity at the crack front is due to an overlapping of the diffraction of the strongly curved regions at the crack front. Thanks to the wide incident spectrum, the tilted crystal regions around the crack front are still in the diffraction condition. Thus, the crack front can be directly visualized as an intense signal line visible at the centre of the diffracted area on the images shown in Fig. 2 ▸. The very short illumination from a single bunch provides a snapshot of the crack front shape, with negligible blurring due to crack movement during exposure. For all the samples, the crack front has a smooth circular shape, within the resolution of our setup, which experimentally confirms for the first time what was previously a working hypothesis for the analysis of crack propagation (Landru *et al.*, 2021 ▸).

### 3.2. Crack front velocity

**Figure 3:**
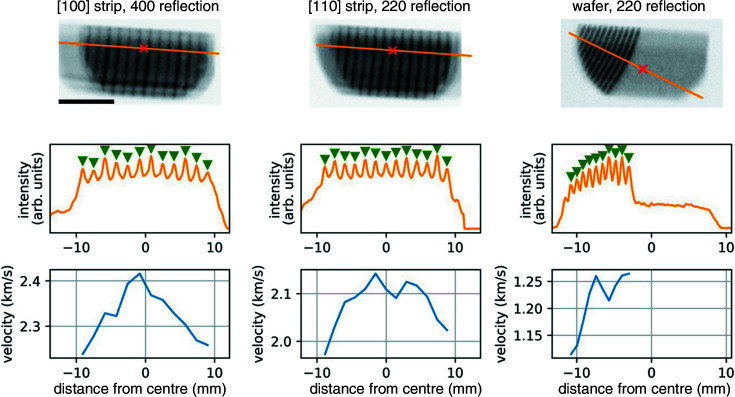
(Top) A single image of all the maximum intensities seen by the camera for the different strip samples and full 300 mm wafer assembly. The centre of the X-ray illuminated area is represented by a red cross and the orange line indicates the position of the line profile extracted below. (Middle) A line profile perpendicular to the crack front. Each peak is due to a single bunch. Space-wise, the exact location of each peak is obtained by a Gaussian fit and indicated by a green triangle. Time-wise, each bunch is 704 ns after the previous one. (Bottom) The evolution of crack front velocity in the X-ray illuminated area, as calculated from the peaks’ space–time positions.

Interestingly, the fracture speed does not seem to depend strongly on the crystal orientation, as the topographs from Figs. 2 ▸(*a*) and 2 ▸(*b*) seem to be quite synchronized. Knowing the horizontal size of the illuminated area (21 ± 1 mm) and the travel time for the crack front through this area (9 ± 1 µs) for Figs. 2 ▸(*a*) and 2 ▸(*b*), the crack front speed can be estimated to be about 2.4 ± 0.4 km s^−1^ for both strip samples. The part of the signal behind the crack front presents several stripes due to the afterglow of the scintillator associated with the pulsed time structure of the X-ray beam in the 4-bunch mode filling pattern of the ESRF storage ring. Indeed, the scintillator is often responsible for a deleterious background intensity between bunches (Rutherford *et al.*, 2016 ▸). Here, we harnessed this time structure to obtain a more accurate instantaneous crack velocity. To do so, we compute a max-filtered image for each sample by taking the maximum value observed at any time for every pixel of our set of images. This is somewhat akin to a ‘bulb exposure’ photograph, showing in a single image all the maximum intensities seen by the camera (Fig. 3 ▸, top row). We then extract a line profile perpendicular to the crack front, showing the peaks due to every single bunch. As mentioned before, the time separation of the bunches is well known and fixed by the storage ring filling pattern. To quantify their space separation, we fitted each peak locally using a Gaussian function to extract the peak position with sub-pixel resolution (Fig. 3 ▸, middle row). We could thus compute the instantaneous crack velocity for each bunch (Fig. 3 ▸, bottom row).

The crack velocity is slightly higher for the strip sample along [100] than for the strip along [110] (2.2–2.4 km s^−1^ and 2.0–2.15 km s^−1^, respectively). This can be explained in the context of dynamic crack propagation, where the asymptotic crack speed *v* is proportional to the Rayleigh wave speed *v*_R_ (Freund, 1990 ▸), 

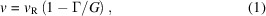

with Γ the fracture energy, related to the area between the microcracks where the fracture must break the material, and *G* the energy release rate, *i.e.* the internal elastic energy recovered when the material is cracked, here directly related to the pressure inside the microcracks (Massy *et al.*, 2015 ▸). Since the strip samples along [110] and along [100] have been cut out from the same wafer, the microcracks are identical (density, internal pressure) and thus Γ and *G* should be identical as well. The Rayleigh speed depends, however, on the propagation direction, from 4.90 km s^−1^ along [100] to 5.06 km s^−1^ along [110] (Pratt & Lim, 1969 ▸). Thus, the observation of a faster crack propagation along [100] is not consistent with this description. We also note that the instantaneous velocity for the 300 mm wafer sample is significantly lower. Both of these results are explained below.

The crack speed is noticeably higher near the centre of the X-ray illuminated area for each strip sample: 2.41 km s^−1^ (2.13 km s^−1^) at the centre versus 2.24 km s^−1^ (1.97 km s^−1^) at the edge of the [100] strip sample ([110] strip sample). This can actually be explained by local heating of the sample due to the intense X-ray beam. As mentioned before, the energy release rate *G* is proportional to the pressure in the microcracks and thus to the temperature. The relation 1 − *v*/*v*_f_ = *A*/*T*, where *A* is a constant, was already verified experimentally in a previous study (Massy *et al.*, 2015 ▸). Therefore, the observed 8% speed increase at the centre of the field of view for both samples corresponds to a 5–6% temperature increase. This would amount to Δ*T* = 15–25 K at temperatures between 300 and 400 K. Such a temperature gradient also results in a small variation in the lattice parameter due to the thermal dilation, which in turn results in a small (opposite) angular variation in the diffraction, and thus a small shift in position on the detector. Considering the lattice expansion coefficient of Si, the corresponding relative error on the velocity determination can be estimated to be below 1%.

Similarly, a different average sample temperature over the field of view can explain the discrepancy in the numerical values between the dynamic model (*v*_110_/*v*_100_ = 1.03) and the experimental value (*v*_110_/*v*_100_ = 0.90 ± 0.01). The faster crack velocity along [100] would correspond to a 13 ± 1% temperature increase compared with the [110] strip, *i.e.* the [100] strip sample was about 40–50 K hotter. A longer alignment time for the first [100] strip sample would have resulted in an increased exposure to the beam, and therefore in increased heating before the fracture was triggered. We note that the samples were too hot to be touched with bare hands right after the measurement, indicating temperatures typically above 340 K.

Using the same approach, comparing the 300 mm wafer sample and the strip samples, the respective velocities indicate an average temperature about 30% higher in the strip samples. This indicates that the strip samples were about 100 K hotter than the wafer sample, which is consistent with their much smaller heat capacity and heat dissipation.

### 3.3. Post-crack oscillations

As shown in Fig. 2 ▸, we observe a signal modulation in the wake of the crack front. This is particularly visible on the edges of the beam footprint. The detector image actually shows the superimposition of two scattered images of the incident beam with a time-dependent shift. These two images are the results of scattering by the two arms of the crack opening. The crack opening angular displacement can thus be obtained from analysis of the diffracted image motions. Given the beam divergence (21 mm over 150 m) and bandwidth (λ/Δλ = 100), we estimate from the Du Mond diagram for the 220 (400) reflection that sample rotations up to about 0.8 (1.1) mrad can still be imaged (see the supporting information). This is larger than the typical deflection that we have previously measured using optical reflection (Ronseaux *et al.*, 2021 ▸), indicating that the full movement of the two sample plates after the fracture can be captured.

**Figure 4:**
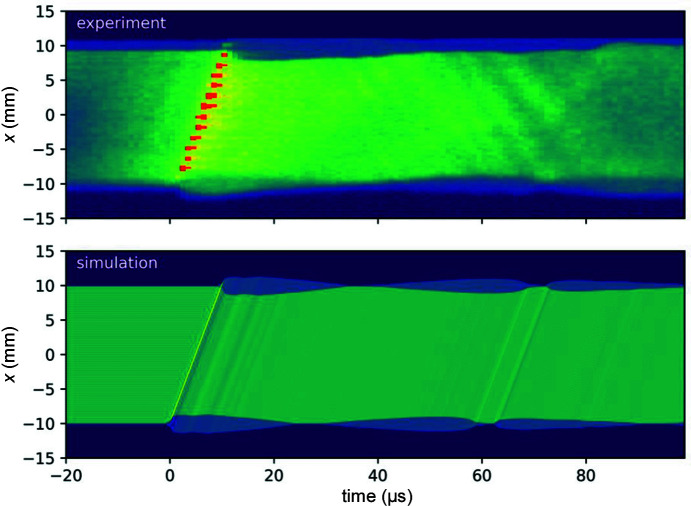
Diffracted intensity for the [110] strip sample as a function of time and position along the length *x* (*i.e.* along [110]), taken at the central *y* position, for (top) experimental data and (bottom) simulated data considering the propagation of the gap opening profile.

To analyse the post-crack sample movement, we extract the centre line of the diffracted image (averaged over five pixels) and plot it as a function of time in Fig. 4 ▸. The fracture propagation from one side to the other is clearly seen as an intense diagonal line with slope *v*_f_. The crack opening is directly visible in the wake of the crack front. Shortly after the crack front crossing, the two separated plates can be seen as the half-intensity areas at each extremity, where only one of the two plates contributes to the diffraction. Note that similar small shadows could be observed on each side before the appearance of the crack, simply due to a slight misalignment of the crystal planes in the two bonded wafers. The angular separation just behind the crack indicates a ‘wedge’ shape of the crack opening. This is consistent with IR interferometry measurements of the crack opening (Massy *et al.*, 2015 ▸) and optical reflection measurements (Ronseaux *et al.*, 2021 ▸). From linear elastic fracture mechanics, the profile is parabolic in the close vicinity of the crack front, as strain varies as *K*/(*x* ^1/2^), with *K* the stress intensity factor. At larger distances, a regular beam bending strain should be expected. The wedge shape is probably due to the transition between these two regimes with opposite curvatures and a convolution with the experimental resolution. The later oscillatory shape finally indicates a back-and-forth movement of the plates.

In order to go further in our analysis, we simulated the diffracted images using the following approach. The IR laser signal to trigger the high-speed camera also provides an indirect measurement of the gap profile behind the crack front. The method is described elsewhere (Massy *et al.*, 2015 ▸) and can be summarized as follows. The opening of the crack creates two partially reflecting surfaces. The transmission through this gap is thus a function of the gap opening, with maxima every λ/2, resulting in fringes in the detected signal as a function of time. It is not directly possible to know if the gap is opening or closing by this quantified amount at each fringe, but it is reasonable to assume that a maximum in the delay between two successive fringes (vanishing speed) indicates a reversal of the motion. We thus obtain the local discrete profile ζ(*t*) of the gap at the location of the IR laser, where each plate is deformed by ± ζ(*t*)/2 (see the supporting information). Assuming that the profile is invariant by translation, *i.e.* the sample deformation behind the crack is the same throughout the crack propagation, ζ(*t*) = ζ(−*x*/*v*_f_). The angle of the surfaces can then be computed as

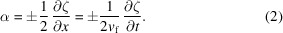




The corresponding diffraction image can be simulated using a simple ray-tracing approach. For a time step *t*, a length of 21 mm of the deformation profile is illuminated by parallel rays every 10 µm. Each ray is then deflected by ± 2α, where α is the local sample rotation defined above. The rays propagate over *d* = 1.7 m and hit the detector, where all the resulting positions are binned according to the pixel size. The initially illuminated area is shifted for the next time step *t* + Δ*t* by *v*_f_Δ*t*. The resulting simulated image is shown in Fig. 4 ▸ as well. The main features are well captured by the simulation, confirming that our description is correct. The observed back-and-forth motion of the two plates visible at the edges (*x* = ±10 mm) is actually due to pneumatic oscillations, as described before (Massy *et al.*, 2015 ▸). Small discrepancies can be observed for the later times, which may indicate that the deformation profile is not exactly invariant by translation, probably because of the finite size of the sample and/or local temperature changes. Finally, back-propagating waves are observed in the experimental data as slightly more intense lines with a negative slope in Fig. 4 ▸, yet they are not reproduced by our simple simulations. These waves could actually be flexural waves, recently observed using optical reflection measurements (Ronseaux *et al.*, 2021 ▸).

## 4. Summary and outlook

As had previously been foreseen (Rack *et al.*, 2016 ▸), X-ray diffraction imaging using a large parallel synchrotron beam has proven to be a unique tool for real-time dynamic fracture studies, thanks to the high flux and temporal resolution of synchrotron illumination, coupled with high-speed cameras. Here, we have shown that synchrotron X-ray diffraction imaging can be fast enough for the *in situ* study of fracture mechanics in a brittle material, at speeds of a few kilometres per second. We have harnessed this technique to tackle the industrially relevant problem of fracture propagation in the Smart Cut technology and to obtain otherwise inaccessible data. In particular, both real-space and real-time images of the crack front propagating in single-crystal Si at speeds near the Rayleigh wave speed have been acquired for the first time. This direct visualization of the crack front shape has experimentally confirmed the homogeneous propagation of the fracture, which was previously only a working hypothesis for the fractography analysis of post-split surfaces.

Thanks to the time structure of the synchrotron source, the local crack velocity could be measured and compared with point measurements using IR laser transmission. While the average velocity value is consistent with IR measurements, the effect of increased speed near the image centre could be interpreted as local heating due to the intense X-ray beam, in agreement with previous studies (Massy *et al.*, 2015 ▸; Landru *et al.*, 2021 ▸).

Finally, the post-split images have shown the movements of the separated wafers and confirmed the importance of pneumatic oscillations in the wake of the fracture. Additional back-propagating waves were also observed, reminiscent of flexural waves and requiring further analysis.

These results open a vast avenue of potential future studies. For example, it could be very interesting to trigger the fracture thermally using the X-ray heating of the sample, to be as close as possible to the phenomenon occurring in the annealing furnace, and thus observe the very first stages of fracture initiation. A more detailed stress and strain description at the crack tip is now probably within reach, using *e.g.* a larger sample-to-detector distance, combined with the improved brilliance of the new ESRF source. Also, coupling high-speed diffraction imaging (a few megahertz) with more conventional yet fast optical imaging (10 to 100 kHz) could provide a full understanding of fracture propagation, the related emission of acoustic waves and their intimate interplay. Lastly, our approach here is not limited to silicon and can be extended to any crystalline material of interest that can be transferred using the Smart Cut technology, such Ge, GaAs or SiC.

## Supplementary Material

Overview of the data acquired during beamtime. DOI: 10.1107/S1600576722006537/vb5040sup1.pdf
