# Supplementary material for: Brittle fracture studied by ultra-high-speed synchrotron X-ray diffraction imaging
Source: J Appl Crystallogr. 2022 Jul 30;55(Pt 4):911–8. doi: 10.1107/S1600576722006537 (PMC9348878; doi:10.1107/S1600576722006537)
Supplement: Supplementary file 1 [file j-55-00911-sup1.pdf]

JOURNAL OF APPLIED CRYSTALLOGRAPHY

Volume 55 (2022)

Supporting information for article:

Brittle fracture studied by ultra-high-speed synchrotron X-ray diffraction imaging

Antoine Petit, Sylvia Pokam, Frederic Mazen, Samuel Tardif, Didier Landru, Oleg Kononchuk, Nadia Ben Mohamed, Margie P. Olbinado, Alexander Rack and Francois Rieutord

Data overview

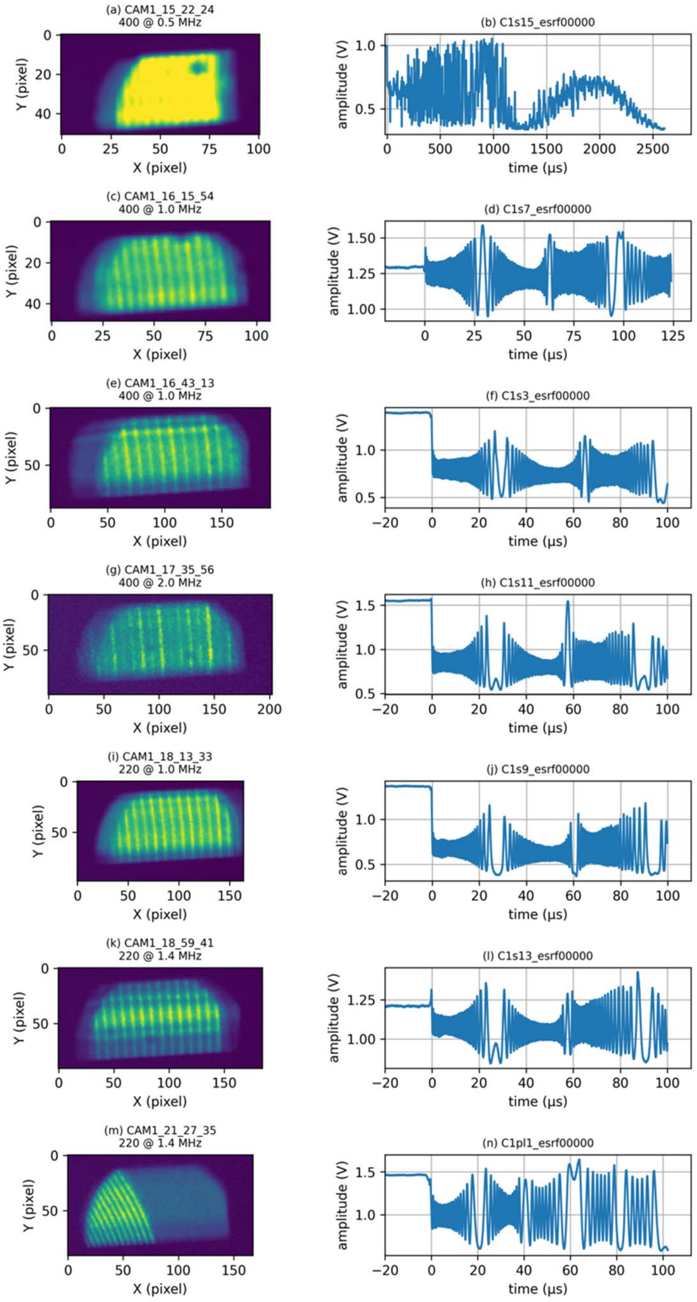

Figure S1: Overview of the data acquired during the beamtime in different samples. (a), (c), (e), (g), (i), (k), (m): Max-filtered image of all the acquired frames. All samples are either [100] or [110] strip samples, except for the last one which was a full wafer. The measured Bragg reflection is indicated in the title of each figure. (b), (d), (f), (h), (j), (l), (n): corresponding oscilloscope signal from the IR laser.

Time evolution in the horizontal direction

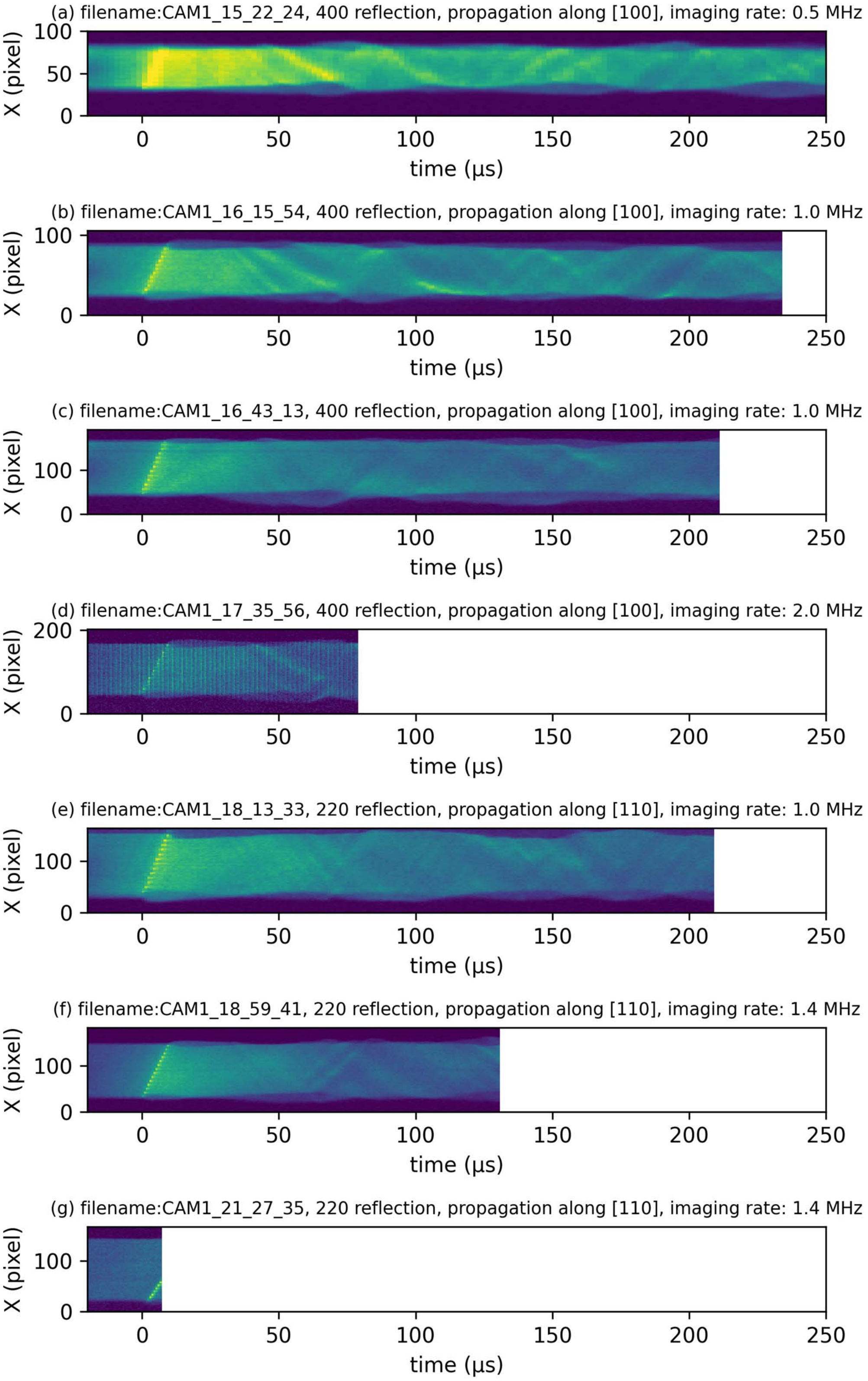

Figure S2: (a)-(g) Time evolution in the horizontal *x* plane for all the samples (see Figure S1).

Du Mond diagram for the ID19 experiment

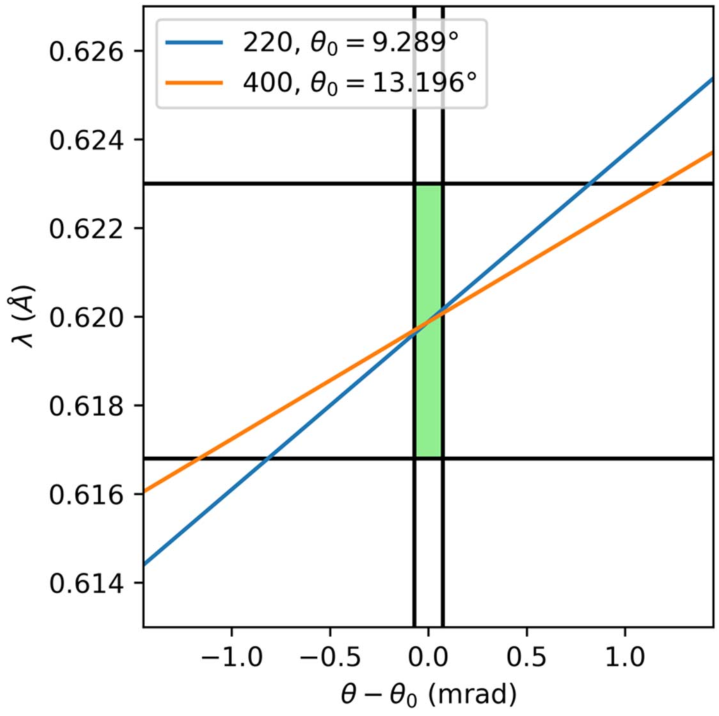

Figure S3: Du Mond diagram for the experiment, for the 220 and 400 reflections of Si at 20 keV. The blue and orange solid lines are the Bragg law for each reflection $\lambda = 2d_{hkl}\, sin\,\theta$, where θ_0 is the nominal value at 20 keV ($\lambda = 0.620$Å) . Any rotation of the sample results in a horizontal shift of the corresponding line. The black vertical (horizontal) lines indicate the estimated divergence (bandwidth) of the beam and delimit the allowed imaging region (green area).

Comparison of the crack velocity in the [110] and [100] strip samples

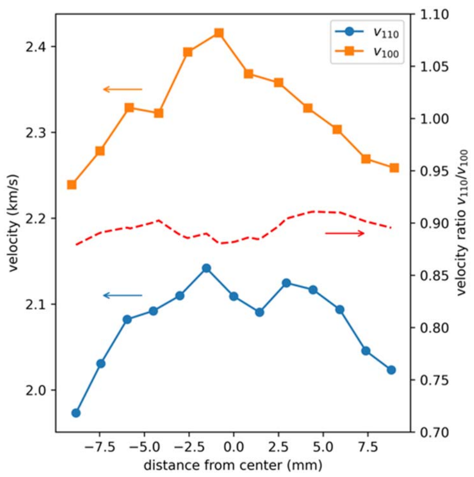

Figure S4: Comparison of the local crack velocity measured in the [110] and [100] strip samples and their ratio.
